# Association between Ambient Temperature and Blood Pressure and Blood Pressure Regulators: 1831 Hypertensive Patients Followed Up for Three Years

**DOI:** 10.1371/journal.pone.0084522

**Published:** 2013-12-31

**Authors:** Qing Chen, Jinwei Wang, Jun Tian, Xun Tang, Canqing Yu, Roger J. Marshall, Dafang Chen, Weihua Cao, Siyan Zhan, Jun Lv, Liming Lee, Yonghua Hu

**Affiliations:** 1 Department of Hygienic Toxicology, College of Preventive Medicine, Third Military Medical University, Chongqing, P. R. China; 2 Department of Epidemiology & Biostatistics, School of Public Health, Peking University Health Science Center, Beijing, P. R. China; 3 Section of Epidemiology and Biostatistics, School of Population Health, University of Auckland, Auckland, New Zealand; University of Maryland School of Medicine, United States of America

## Abstract

Several studies have suggested an association between ambient air temperature and blood pressure. However, this has not been reliably confirmed by longitudinal studies. Also, whether the reaction to temperature stimulation is modified by other factors such as antihypertensive medication is rarely investigated. The present study explores the relationship between ambient temperature and blood pressure, without and with antihypertensive medication, in a study of 1,831 hypertensive patients followed up for three years, in two or four weekly check ups, accumulating 62,452 follow-up records. Both baseline and follow-up blood pressure showed an inverse association with ambient temperature, which explained 32.4% and 65.6% of variation of systolic blood pressure and diastolic blood pressure (*P*<0.05) respectively. The amplitude of individual blood pressure fluctuation with temperature throughout a year (a 29 degrees centigrade range) was 9.4/7.3 mmHg. Medication with angiotensin converting enzyme inhibitor benazepril attenuated the blood pressure fluctuation by 2.4/1.3 mmHg each year, though the inverse association of temperature and blood pressure remained. Gender, drinking behavior and body mass index were also found to modify the association between temperature and diastolic blood pressure. The results indicate that ambient temperature may negatively regulate blood pressure. Hypertensive patients should monitor and treat blood pressure more carefully in cold days, and it could be especially important for the males, thinner people and drinkers.

## Introduction

For hypertensive patients, optimal control of blood pressure (BP) helps maintain low and stable level of BP, and may decrease the risk of adverse events, including stroke and heart failure [1]–[5]. Therefore, it is important to understand the factors that regulate the fluctuation of BP. Temperature is suspected to be one of these factors, and several studies have suggested the association of fluctuation of BP with seasons or ambient temperature [6]–[8]. However, most were cross-sectional studies or follow-up studies with few repeated measurements. Except during pregnancy [9], it is unclear whether and to what level, systolic blood pressure (SBP) or diastolic blood pressure (DBP), or both, change with temperature in long-term longitudinal studies. Also, it is unclear whether regulators of hypertension, such as age, smoking behavior and antihypertensive medication, modify the association of BP and temperature. The aim of the present research is to investigate the association between BP and ambient temperature, and further explore potential factors that would modify this association. It is based on a three-year surveillance of 1,831 hypertensive patients with 62,452 repeated measurements.

## Materials and Methods

### Ethics Statement

This study was approved by the institutional review boards of Peking University. All subjects provided written informed consent.

### Study Design and Participants

The research is a secondary analysis of a longitudinal study with 3-year follow-up of 1,831 hypertensive patients, which was a part of the Chinese Community-based Comprehensive Prevention and Control of Hypertension (CCPACH), a project consisting of 34,770 permanent residents over 35 years old in the Nanshi District, Shanghai, China [10]. The subjects were recruited from a census of BP in the residents from June, 1997 to April, 1998. In brief, among the 34,770 participants, those with mean BP≥140/90 mmHg or current use of antihypertensive medication were defined as hypertensive patients, After exclusion of patients with severe morbidity (recent myocardial infarction, stroke, uncontrolled angina within the past 3 months, severe liver or renal disease), 1831 patients with written informed consent were recruited into the three-year surveillance of BP. Urine samples were collected for clinical examinations including urine protein analysis. Baseline information including age, sex, BMI, and lifestyle (smoking, alcohol consumption), was obtained by a questionnaire. Patients were asked to come back to the clinics of their community for interview every 2 to 4 weeks (every 2 weeks in the first 12 weeks, and every 4 weeks in the later period).

**Figure 1:**
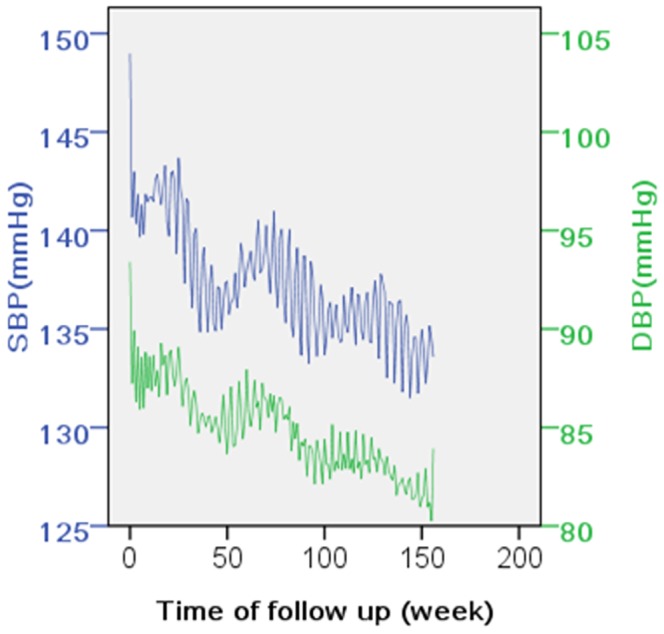
Blood pressure of CCPACH project participants during three-year following up. As a part of the CCPACH project, 1,831 hypertensive patients were prescribed with benazepril and followed up for three years. The patients’ blood pressure declined quickly at the first two weeks, and then began to fluctuate but decrease gradually each year. SBP indicates systolic blood pressure; DBP, diastolic blood pressure.

During baseline and the following interviews, BP was measured in the right arm of seated participants after a 5-minute rest by mercury sphygmomanometer with appropriately sized cuff. Measurement was performed at three 1-minute intervals according to standard protocol [10]. The mean of the three measurements was calculated as the record of BP. At each visit, benazepril hydrochloride, a type of angiotensin converting enzyme inhibitor (ACEI) was prescribed for all patients and the drug dose was adjusted in the first 8 weeks according to whether the participants’ BP was below 140/90 mmHg; a small proportion of patients (57 out of 1831 persons, 3.1%) also received dihydrochlorothiazide. Additional details of study design were reported elsewhere [10], [11]. The average baseline blood pressure of the study population was 149.0/93.4 mmHg right before prescription of benazepril. At the two-week’s follow-up, patients’ BP dropped to 142.9/89.9 mmHg. Since the fourth week, BP declined slowly to 133.2/82.1 mmHg at the 156^th^ week (Figure 1). At the 156^th^ week (three years after recruitment, the end of the surveillance plan), 72.5% of the patients (1,329 persons) remained in the study, with an accumulation of 62,452 follow-up records, of which 60,463 were at the fourth week and after.

### Ambient Temperature

Daily ambient temperatures (highest temperature, lowest temperature, average temperature, and diurnal temperature range) were monitored at Hongqiao airport meteorological station, Shanghai, which was about 16 kilometers away from the clinics and the patients’ residence. Data from June 1997 to April 2001 were retrieved to cover the entire follow-up period.

### Statistical Analysis

The dataset was divided into a baseline (before the prescription of benazepril) and a follow-up dataset. Linear regression was used to investigate the association between temperature and baseline BP, adjusted for gender, age, body mass index (BMI), urine protein, smoking behavior and drinking behavior. Multilevel modeling was implemented to analyze the association between the temperature and the repeated measurement data of the follow-up dataset. Random effects for the duration of medication and intercept were included in the model. The covariance structure was defined as unstructured, and the estimation method was maximum likelihood. Besides the covariates mentioned above, the baseline BP and the medication duration was also included. Interactions of temperature and other covariates were examined as product terms.

To estimate the contribution of temperature to the average change of BP in aggregated weeks, the association between the ambient temperature and the average weekly BP was examined by linear regression. The average BP of the subjects who were recruited in the same week was calculated, so was the mean of temperature. Multiple correlation coefficients (R^2^) were used to indicate the proportion of variance that could be explained by ambient temperature; the R^2^ of medication duration was also investigated.

As BP dropped quickly in the first few weeks of benazepril medication and more slowly in the later period, association analyses were conducted only with BP records from the 4^th^ week to the 156^th^ week (60,463 interviews, 33 records for each participant on average). To exclude the possible bias from the intake of dihydrochlorothiazide, the analyses were repeated after the 57 patients involved were dropped.

The statistical analyses were performed in SPSS for windows (version 15.0, SPSS Inc., Chicago, Illinois; for linear regression analyses, paired t-test, repeated measure analyses of variance and figure production) and SAS (version 9.1.3, SAS Institute Inc., Cary, North Carolina; for multilevel model analyses).

## Results

### Ambient Temperature and Individual Blood Pressure

Table 1 shows the baseline characteristics of the participants. It shows that other than smoking and drinking, males and females were similar with respect to their blood pressure and BMI.

**Table 1:** Baseline characteristics of the participants.

Characteristics	Male (n = 1,090)	Female(n = 741)
Age(year)	55.3±10.5	55.2±9.5
SBP(mmHg)	148.9±15.0	149.0±15.2
DBP(mmHg)	94.3±8.6	92.1±7.9
BMI	24.5±2.8	24.5±3.4
Smoking behavior (%)		
Never smoke	47.9	98.0
Ever smoked	12.5	0.5
Smoking	39.6	1.5
Drinking behavior (%)		
Not drinking	89.4	99.7
<100 g/day	7.1	0.1
≥100 g/day	3.5	0.1
Urine protein (%)		
−	81.8	78.7
±	8.6	7.8
+	7.2	10.5
++	1.7	2.4
+++	0.7	0.5

Continuous variables were described as mean ± standard deviation. Drinking behavior was recorded as not drinking, drinking <100 g wine per day, or drinking ≥100 g wine per day. SBP indicates systolic blood pressure; DBP, diastolic blood pressure; BMI, body mass index.

The relationship between ambient temperature and BP was examined with the baseline and follow-up datasets of individual BP records. As expected, daily average temperature was significantly associated with baseline SBP and DBP (*β* = −0.266 in SBP model, 95% CI: −0.352 to −0.181, *P*<0.001; *β* = −0.173 in DBP model, 95% CI: −0.220 to −0.126, *P*<0.001) after adjustment for gender, age, BMI, urine protein, smoking behavior and drinking behavior. Those associations were also significant in the individual follow-up analysis (*β* = −0.214 in SBP model, 95% CI: −0.222 to −0.206, *P*<0.001; *β* = −0.144 in DBP model, 95% CI: −0.149 to −0.139, *P*<0.001) in which multilevel models were used and baseline BP and medication duration were additionally adjusted. The average amplitude of temperature change in the follow-up years was 29.0 degrees (2.3 to 31.2 centigrade), so the average amplitude of BP fluctuation each year with ambient temperature was estimated to be 6.2/4.2 mmHg. The highest and lowest temperature was also examined, showing quite similar results (data not shown). There was no significant association between diurnal temperature range and SBP or DBP. Hence, only daily average temperature was chosen to represent the effect of ambient temperature in the follow-up analyses.

### Regulators of Blood-pressure Response to Temperature Change

The interactions between daily temperature and other factors were also investigated. In the SBP model, the temperature and medication duration interaction, as well as the interaction of temperature and age, was statistically significant (Table 2). The regression coefficient of the medication-temperature interaction was 0.0016 (*P*<0.001), so under the benazepril therapy, the reaction of SBP to the change of ambient temperature (29.0 degrees throughout a year) was estimated to decrease by 2.4 mmHg each year. To confirm these interactions, medication duration and age were transformed into ordinal categories and the follow-up dataset was stratified into subsets in which the effects of daily average temperature on BP were estimated separately. In that process, there seemed to be a linear trend in the medication categories but not for the age categories (Table S1 and Table S2). As the medication of benazepril continued (from first to third year), the impact of temperature on SBP decreased. The fluctuation of SBP with temperature in the three years was 11.9, 8.1 and 4.9 mmHg respectively.

**Table 2:** Association of BP and ambient temperature as well as other factors: derived from the 60,463 records.

Factors	SBP				DBP			
	*β*	Lower	Upper	*P* value	*β*	Lower	Upper	*P* value
Baseline SBP^a^	0.240	0.219	0.262	<0.0001	–	–	–	–
Baseline DBP^b^	–	–	–	–	0.299	0.274	0.323	<0.0001
Temperaturea,b	−0.258	−0.302	−0.213	<0.0001	−0.252	−0.303	−0.201	<0.0001
Medication durationa,b	−0.073	−0.078	−0.069	<0.0001	−0.053	−0.056	−0.051	<0.0001
Gendera,b	−1.337	−2.070	−0.605	0.0003	−1.228	−1.682	−0.775	<0.0001
Age^a^	0.196	0.161	0.230	<0.0001	−0.013	−0.034	0.007	0.2045
BMIa,b	0.191	0.089	0.294	0.0002	0.075	0.003	0.147	0.0414
Drinking behavior	0.158	−1.156	1.472	0.8141	0.615	−0.301	1.531	0.1879
Smoking behavior^a^	1.126	0.346	1.905	0.0046	−0.013	−0.034	0.007	0.1982
Urine proteina,b	3.505	1.541	5.469	0.0005	1.939	0.669	3.209	0.0028
Medication duration*temperaturea,b	0.002	0.001	0.002	<0.0001	0.001	0.001	0.001	<0.0001
Gender*temperature^b^	0.013	−0.003	0.028	0.1186	0.024	0.013	0.034	<0.0001
Age*temperature^a^	−0.001	−0.002	0.000	0.0019	0.000	−0.001	0.000	0.3829
BMI*temperature^b^	0.001	−0.002	0.003	0.6691	0.002	0.001	0.004	0.0104
Drinking behavior*temperature^b^	−0.015	−0.047	0.017	0.3664	−0.035	−0.056	−0.013	0.0014
Smoking behavior*temperature	−0.003	−0.019	0.014	0.7567	−0.009	−0.021	0.004	0.1715
Urine protein*temperature	0.031	−0.019	0.081	0.2296	−0.007	−0.040	0.025	0.6591

The association of daily average ambient temperature and blood pressure were examined with multilevel model, adjusted for other factors listed in the table. SBP indicates systolic blood pressure; DBP, diastolic blood pressure; BMI, body mass index.

^a^ Significant in the SBP model.

^b^ Significant in the DBP model.

In the DBP model, similar medication-temperature interactions existed. The gender-temperature, drinking-temperature, and BMI-temperature interactions were all significant. The regression coefficient of the medication- temperature interaction was 0.00084 (*P*<0.001), so the change in DBP to the ambient temperature change (29 degrees over a year) was estimated to decrease by 1.3 mmHg each year. When stratified by medication duration (in unit of year) the effect of temperature also decreased. The fluctuation of DBP was 7.8, 6.4 and 4.4 mmHg in each year. For males, this fluctuation was 8.4, 7.0 and 4.4 mmHg, while for females it was 7.2, 5.5 and 4.4 mmHg (Table S2, *P*<0.05). The temperature regression coefficients are smaller in the higher BMI group (assigned according to the body mass index reference norm for Chinese adults [12]:<18.5, 18.5 to 23.9, 24.0 to 27.9 and ≥28.0; see Table S1). However, this was not replicated in another confirmation analysis (Table S2). The drinkers’ DBP fluctuation was estimated to be higher than non-drinkers’ (10.8 mmHg vs. 8.4 mmHg, see Table S1), and the difference remained in each of the three years (Table S2).

After these interactions were adjusted (the age-temperature interaction was not included in the SBP model), the regression coefficients of daily average temperature were −0.325 (in SBP model, 95% CI: −0.339 to −0.311, *P*<0.001) and −0.252 (in DBP model, 95% CI: −0.303 to −0.201, *P*<0.001) respectively, which meant a 9.4/7.3 mmHg increase in BP as the ambient temperature decreased by 29.0°C in a year.

### Contribution of Temperature to the Weekly Average BP

**Figure 2:**
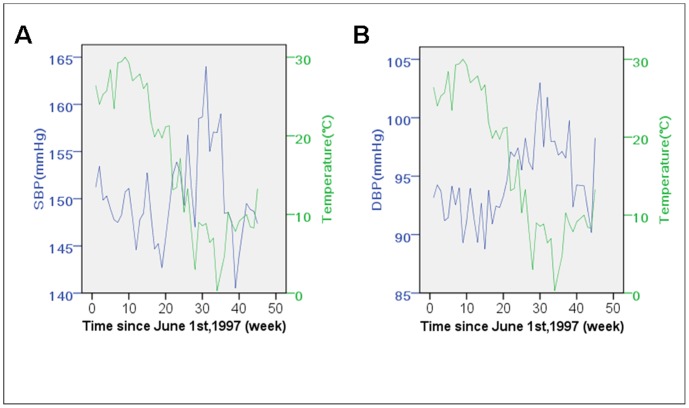
Baseline blood pressure of patients recruited at different time, as well as average ambient temperature. The participants of the study were recruited June,1997 to April,1998. The baseline blood pressure of the participants recruited at different time varied, both systolic (A) and diastolic blood pressure (B) inversely correlated with the ambient temperature, which changed during the recruiting period (*P*<0.05 respectively). SBP indicates systolic blood pressure; DBP, diastolic blood pressure.

Linear regression was used to investigate the association between the average baseline BP of patients recruited in the same week and the mean of weekly ambient temperature (Figure 2). Significant inverse correlations were found for SBP, as well as DBP. The average temperature explained 32.4% (*β* = −0.171, 95% CI: −0.325 to −0.016, *P* = 0.032) and 65.6% (*β* = −0.241, 95% CI: −0.328 to −0.155, *P*<0.001) of the variation of SBP and DBP, respectively.

**Figure 3:**
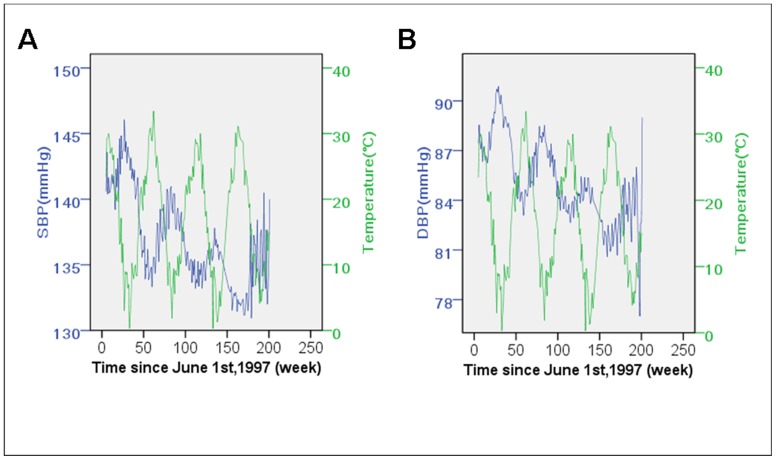
Follow-up blood pressure and the average ambient temperature. The participants were followed up for three years under the medication of benazepril. The average systolic (A) and diastolic blood pressure (B) calculated with the interviews done each week since June 1st, 1997, when the first participant was recruited, was inversely associated with average ambient temperature at the same week (*P*<0.001 respectively). The ambient temperature explained 39.2% and 39.0% of the systolic and diastolic blood pressure variation, and when combined with medication of benazepril, 86.5% and 88.4% could be explained. SBP indicates systolic blood pressure; DBP, diastolic blood pressure.

To investigate the BP fluctuation of the same subjects as temperature changes, the temperature-BP relationship was also examined in the three-year follow-up dataset (Figure 3). Similar linear regression was implemented except that duration of benazepril medication was included to represent the effect of antihypertensive therapy. In the follow-up models without duration of benazepril medication, the average temperature accounted for 39.2% (*β* = −0.189 in SBP model, 95% CI: −0.219 to −0.159, *P*<0.001) and 39.0% (*β* = −0.134 in DBP model, 95% CI: −0.154 to −0.115, *P*<0.001) of BP variation. The full follow-up models (including temperature and medication duration) explained 86.5% and 88.4% of SBP and DBP variation.

**Figure 4:**
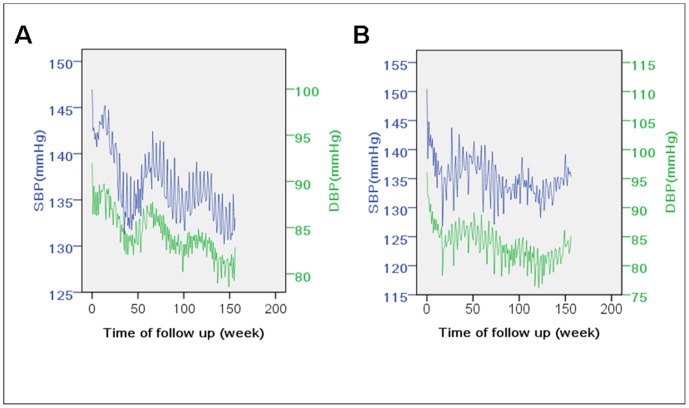
Follow-up blood pressure of patients recruited at around October and February. The blood pressure fluctuation throughout the follow-up period was investigated separately in patients who were recruited in different seasons. Patients recruited at around October (A) and February (B) represented opposite patterns. At about 50^th^, 100^th^, 150^th^ week, the October group achieved the bottom of blood pressure fluctuation, while the February group just rose to its peak. October and February were the turning points of ambient temperature in the area. SBP indicates systolic blood pressure; DBP, diastolic blood pressure.

To further confirm the fluctuation of BP with temperature, subsets of patients recruited around October (from September 1, 1997 to October 31, 1997, n = 716) and February (from December 1, 1997 to April 17, 1998, n = 199; the time span of this group was wider because fewer patients were recruited in that period each day), when the ambient temperatures were highest and lowest in the year of recruitment, were selected separately to investigate the trends. These two groups showed opposite BP trends (Figure 4): at around the 50^th^, the 100^th^ and the 150^th^ week, BP of the first group reached its lowest, while the second group was at its highest.

To exclude the potential influence of dihydrochlorothiazide on the results, the analyses were repeated in a dataset without the 57 subjects involved, and the results did not appreciably change (data not shown).

## Discussion

Based on 1,831 patients with 3-year follow-up, the present research suggests that BP is negatively associated with ambient temperature throughout the year both before and after starting antihypertensive medication (Figure 2 and Figure 3), and no association was found between BP and diurnal temperature range.

If ambient temperature has an effect on BP, a change in ambient temperature may lead to an increase in the incidence of hypertension, and raise public health concerns. Also, it may help explain the increased mortality of cardiovascular diseases in cold seasons [13]. It has long been noted that BP varies in different seasons, and some studies have been conducted to examine whether temperature directly plays a role in that phenomenon. Modesti et al. investigated the relationship between air temperature and daytime BP in the subjects referred to their clinics and found inverse association [7]. Madsen et al. also reported that daily temperature was inversely related to SBP and DBP in a population of Oslo citizens [14]. These studies have large samples, but a limitation is their cross-sectional study design, which precludes investigation of fluctuations of BP in the same subjects.

However, Alpérovitch et al. reported a large longitudinal study with a 2-year follow-up, with interviews made quarterly. Although they found significant differences between BP under the highest and lowest quintile of temperature, they could not address whether there was a threshold above which this fluctuation occurs [15]. Our longitudinal records of BP with an average of 33 interviews for each participant in 3 years may help to overcome the limitations of the previous studies and provide evidence of the effect of ambient temperature on BP. Also, there was no association between BP and diurnal temperature range, suggesting that it is the temperature itself that is related to BP, not the range. Nevertheless, whether a large diurnal range causes fluctuation of BP in a day remains to be investigated.

Hozawa et al. reported that an inverse association between outside temperature and BP was only observed in warmer seasons in Japan [16]. The authors suggested this could be the result of heaters used in cold seasons. This phenomenon was not seen in our study. The possible explanation is that heating was not so commonly used ten years ago in China, so that our data may represent the actual relationship of BP and ambient temperature. No information about the usage of air conditioning was collected in our study. Although the average household ownership rate of air conditioning in China was not so high as it is today. Air-conditioning may therefore reduce the reliability of the ambient temperature’s effect. Portable tracking device of temperature would be an optimal solution for future studies.

We also investigated there are factors that modify the association of ambient temperature and BP. Alpérovitch examined the interaction between antihypertensive medication and temperature, but their subjects were taking several kinds of drugs and the researchers did not find significant differences [15]. In our study, we found that the BP fluctuation with temperature decreased gradually as benazepril medication continued. The amplitude of BP sensitivity to cold stimulation has long been linked to the risk of hypertension [17], [18]. Hence treatment of hypertension may in turn improve the homeostasis of BP under temperature change. Cold stimulation can induce α-adrenergic vasoconstriction and increase total peripheral resistance, initiated with mitochondrial reactive oxygen species activation [19], and ACEI was found to counteract this effect [20], [21]; furthermore, researchers have suggested that the cold pressor test blunted flow-mediated dilation of brachial arteries, whereas ACEI attenuated the impaired response in hypertensive patients [22]–[24]. This might be the underlying mechanism of the interactions observed in our study but further validation is needed.

Some researchers have reported that older people’s BP is more sensitive to temperature change [15], [25], [26]. We also found significant age-temperature interaction in multilevel model analysis of SBP. However this was not confirmed by an alternative stratified analysis, so it is uncertain whether the interaction really exists. Another study on this topic has reported null association [27], and another study showing a significant interaction is unreliable due to small sample size [7]. The difference of BP reaction to temperature at different ages still needs confirmation by future researches.

Unlike some reports which suggested that women are more sensitive to cold stimulation than men [28]–[30], our results showed that men experienced greater BP fluctuation when the ambient temperature changed. The effect of gender difference on BP reaction still seems controversial, even though there is some evidence which supports the more vulnerable status of males [31], [32]. The present study may provide new evidence, but further confirmation by future studies is needed.

Drinking behavior is another potential effect modifier of temperature-BP association. The drinkers’ DBP fluctuated slightly more than the non-drinkers’ DBP as ambient temperature changed. This may result from the effect that alcohol increases superficial blood flow and emission of heat, leading to higher sensitivity to ambient temperature stimulation. Drinking behaviors of the subjects were primarily recorded as no drinking, drinking less than 100 g wine per day, or drinking at least 100 g per day in the study. A “J” curve of the effect of drinking was once suspected for the association between drinking and BP or coronary heart disease [33]–[35]. However, in the present study we did not find this pattern on the temperature-BP association. The effect of temperature was similar in the two drinking groups (*P* = 0.655, so we combined them together) and higher than the non-drinking group.

We also found that BMI may modify the temperature-BP association. We found people with higher BMI seemed to experience a smaller fluctuation of DBP with ambient temperature. Generally, BMI was not consistent with our original hypothesis that obese people who often had worse cardiovascular function had to face more difficulties in adapting to environmental stimulations. Possibly people with more fat maintained their body temperature more easily, so feel milder stimulation of cold. This was supported by Kingma’s study which reported that large body fat proportion help protect against the adverse effect of cold [26]. It indicates that thinner people should perhaps try to keep warm during cold days so as to minimize the BP fluctuation. On the other hand, the results also imply that obese patients’ BP may remain high in hot weather without effective therapy [36].

Temperature has been reported to be associated with mortality in a study with over 169,000 clinic visits [37]. Hence the identification of vulnerable individuals may help to reduce the mortality and the medical cost in the population. Most effect modifiers of temperature-BP relationship that we found have only a mild effect. However, if these were real, patients with combination of the risk factors may need improved control of their BP. These findings need to be confirmed by further research.

Our data was collected a decade ago so ambient temperature during that period do not necessarily reflect the current conditions. But since our aim is the relationship of blood pressure and temperature, whether the data is old seems of secondary importance. Nevertheless, it would be meaningful to study the health effects of the temperature in recent years as the climate today may be somewhat different to that of ten years ago.

In summary, the present research suggests that ambient temperature appeared to affect BP. Medication with ACEI benazepril may help to keep BP stable when ambient temperature changes. Thinner male patients who drink may need to monitor and treat BP more rigorously. The findings of the present research may have implications for clinical management of hypertension.

## Supporting Information

Table S1 **Stratified analyses of temperature-BP association (regression coefficients).** The significant interactions found in multilevel models were further examined by this method: the regression coefficient of temperature was calculated separately in each stratum (divided by the factor suspected to interact with temperature) and the regression coefficients among different stratum were compared by examining the confidence intervals for their difference with the following formula: where β_1_ and β_2_ were the regression coefficients of temperature, and SE_1_ and SE_2_ were their respective standard errors.
(DOC)

Table S2 **Stratified analyses of temperature-BP association (sensitivity).** Besides the examination of overlap between confidence intervals (Table S1), another method was used to confirm the interactions: in each year during follow-up, the two time points at which BP of the population reached its peak or nadir were selected to calculate the BP difference and temperature difference between them, and “sensitivity” was calculated as the ratio of the BP difference to the temperature difference. Except the age-temperature interaction, all the interactions found in multilevel models showed significance in at least one of the two analyses (Table S1 or Table S2).
(DOC)
