# Correction: Association between Ambient Temperature and Blood Pressure and Blood Pressure Regulators: 1831 Hypertensive Patients Followed Up for Three Years

**DOI:** 10.1371/journal.pone.0091604

**Published:** 2014-02-27

**Authors:** The PLOS ONE Staff

Supporting Information Tables 1-2 contain track changes. The correct, unmarked versions of Tables S1-S2 can be viewed below.

## Supporting Information

Table S1 **Stratified analyses of temperature-BP association (regression coefficients).** The significant interactions found in multilevel models were further examined by this method: the regression coefficient of temperature was calculated separately in each stratum (divided by the factor suspected to interact with temperature) and the regression coefficients among different stratum were compared by examining the confidence intervals for their difference with the following formula: where β1 and β2 were the regression coefficients of temperature, and SE1 and SE2 were their respective standard errors.
(TIF)

Table S2 **Stratified analyses of temperature-BP association (sensitivity).** Besides the examination of overlap between confidence intervals (Table S1), another method was used to confirm the interactions: in each year during follow-up, the two time points at which BP of the population reached its peak or nadir were selected to calculate the BP difference and temperature difference between them, and “sensitivity” was calculated as the ratio of the BP difference to the temperature difference. Except the age-temperature interaction, all the interactions found in multilevel models showed significance in at least one of the two analyses (Table S1 or Table S2).
(TIF)
