# Supplementary material for: Association between Ambient Temperature and Blood Pressure and Blood Pressure Regulators: 1831 Hypertensive Patients Followed Up for Three Years
Source: PLoS One. 2013 Dec 31;8(12):e84522. doi: 10.1371/journal.pone.0084522 (PMC3877276; doi:10.1371/journal.pone.0084522)
Supplement: Table S1 — Stratified analyses of temperature-BP association (regression coefficients). The significant interactions found in multilevel models were further examined by this method: the regression coefficient of temperature was calculated separately in each stratum (divided by the factor suspected to interact with temperature) and the regression coefficients among different stratum were compared by examining the confidence intervals for their difference with the following formula: where β1 and β2 were the regression coefficients of temperature, and SE1 and SE2 were their respective standard errors. (DOC) [file pone.0084522.s001.doc]

Table S1. Stratified analyses of temperature-BP association (regression coefficients)

| Model | Stratifications | | *β* | Fluctuation (mmHg) ^a^ | *P* value | Intra-  stratum  significance ^b^ |
| --- | --- | --- | --- | --- | --- | --- |
| SBP | Medication duration (week) |  |  |  |  |  |
|  |  | 4 to 52 | -0.2363 | 6.9 | <0.0001 |  |
|  |  | 53 to 104 | -0.0862 | 2.5 | 0.0371 | 0.0069 |
|  |  | 105 to 156 | -0.0405 | 1.2 | 0.2584 | 0.0001 |
|  | Age(year) |  |  |  |  |  |
|  |  | <50 | -0.3237 | 9.4 | <0.0001 |  |
|  |  | 50 to 54 | -0.3138 | 9.1 | <0.0001 | 0.5955 |
|  |  | 55 to 59 | -0.3585 | 10.4 | <0.0001 | 0.0913 |
|  |  | 60 to 64 | -0.3013 | 8.7 | <0.0001 | 0.3608 |
|  |  | 65 to69 | -0.3003 | 8.7 | <0.0001 | 0.4193 |
|  |  | ≥70 | -0.3528 | 10.2 | <0.0001 | 0.3182 |
| DBP | Medication duration (week) |  |  |  |  |  |
|  |  | 4 to 52 | -0.2658 | 7.8 | <0.0001 |  |
|  |  | 53 to 104 | -0.1574 | 4.8 | 0.0019 | 0.1017 |
|  |  | 105 to 156 | -0.1286 | 3.6 | 0.0038 | 0.0257 |
|  | Gender |  |  |  |  |  |
|  |  | Male | -0.2434 | 7.1 | <0.0001 |  |
|  |  | Female ^c^ | -0.1877 | 5.4 | <0.0001 | 0.1008 |
|  | BMI |  |  |  |  |  |
|  |  | <18.5 | -0.0910 | 2.6 | 0.3988 |  |
|  |  | 18.5 to 23.9 | -0.2425 | 7.2 | <0.0001 |  |
|  |  | 24.0 to 27.9 | -0.1847 | 5.4 | <0.0001 | 0.0798 |
|  |  | ≥28.0 | -0.1032 | 3.0 | 0.0446 | 0.0134 |
|  | Drinking behavior |  |  |  |  |  |
|  |  | Not drinking | -0.2898 | 8.4 | <0.0001 |  |
|  |  | Drinking | -0.3725 | 10.8 | 0.0048 | 0.5367 |

When interactions were found between daily average ambient temperature and other continuous variables, these factors were transformed into ordinal categories, and regression coefficients (*β*) of temperature were calculated respectively in the stratified datasets, adjusted for other significant variables in the whole-dataset analyses. BP indicates blood pressure; SBP, systolic blood pressure; DBP, diastolic blood pressure; BMI, body mass index.

^a^ Estimated by multiplying the absolute value of the regression coefficient by 29, which was the yearly average temperature change.

^b^ Regression coefficient in each stratum (except the first one) was compared to the first stratum by examining the overlap between the confidence intervals and *P* values were shown. The BMI stratums were compared to the second one (the “18.5 to 23.9” group) as the regression coefficient in the first stratum was not significant.

^c^ Adjusted for baseline DBP, temperature, medication duration and medication- temperature interaction as no other variables were significant in the regression.

|  |  | |  | | |  | | |  |
| --- | --- | --- | --- | --- | --- | --- | --- | --- | --- |
|  |  | |  |  |  |  |  |  |  |
|  |  | |  |  |  |  |  |  |  |
|  |  |  |  |  |  |  |  |  |  |
|  |  |  |  |  |  |  |  |  |  |
|  |  |  |  |  |  |  |  |  |  |
|  |  | |  |  |  |  |  |  |  |
|  |  |  |  |  |  |  |  |  |  |
|  |  |  |  |  |  |  |  |  |  |
|  |  |  |  |  |  |  |  |  |  |
|  |  |  |  |  |  |  |  |  |  |
|  |  |  |  |  |  |  |  |  |  |
|  |  |  |  |  |  |  |  |  |  |
|  |  | |  |  |  |  |  |  |  |
|  |  |  |  |  |  |  |  |  |  |
|  |  |  |  |  |  |  |  |  |  |
|  |  |  |  |  |  |  |  |  |  |
|  |  | |  |  |  |  |  |  |  |
|  |  |  |  |  |  |  |  |  |  |
|  |  |  |  |  |  |  |  |  |  |
|  |  | |  |  |  |  |  |  |  |
|  |  |  |  |  |  |  |  |  |  |
|  |  |  |  |  |  |  |  |  |  |
|  |  |  |  |  |  |  |  |  |  |
|  |  |  |  |  |  |  |  |  |  |
|  |  | |  |  |  |  |  |  |  |
|  |  |  |  |  |  |  |  |  |  |
|  |  |  |  |  |  |  |  |  |  |
