# Supplementary material for: Association between Ambient Temperature and Blood Pressure and Blood Pressure Regulators: 1831 Hypertensive Patients Followed Up for Three Years
Source: PLoS One. 2013 Dec 31;8(12):e84522. doi: 10.1371/journal.pone.0084522 (PMC3877276; doi:10.1371/journal.pone.0084522)
Supplement: Table S2 — Stratified analyses of temperature-BP association (sensitivity). Besides the examination of overlap between confidence intervals (Table S1), another method was used to confirm the interactions: in each year during follow-up, the two time points at which BP of the population reached its peak or nadir were selected to calculate the BP difference and temperature difference between them, and “sensitivity” was calculated as the ratio of the BP difference to the temperature difference. Except the age-temperature interaction, all the interactions found in multilevel models showed significance in at least one of the two analyses (Table S1 or Table S2). (DOC) [file pone.0084522.s002.doc]

Table S2. Stratified analyses of temperature-BP association (sensitivity)

| BP | Stratifications | | Sensitivity(mmHg/℃) | | | Fluctuation(mmHg)^a^ | | | *P* value ^b^ |
| --- | --- | --- | --- | --- | --- | --- | --- | --- | --- |
|  |  | | Year1 | Year2 | Year3 | Year1 | Year2 | Year3 |  |
| SBP | Medication duration | |  |  |  |  |  |  |  |
|  |  | 29th vs.59th week | -0.41 | - | - | 11.89 | - | - | <0.0001 |
|  |  | 84th vs.112th week | - | -0.28 | - | - | 8.12 | - | <0.0001 |
|  |  | 133rd vs.163th week | - | - | -0.17 | - | - | 4.93 |  |
|  | Age(year) | |  |  |  |  |  |  | 0.0553 |
|  |  | <50 | -0.39 | -0.21 | -0.17 | 11.31 | 6.09 | 4.93 |  |
|  |  | 50 to 54 | -0.39 | -0.25 | -0.17 | 11.31 | 7.25 | 4.93 |  |
|  |  | 55 to 59 | -0.44 | -0.35 | -0.17 | 12.76 | 10.15 | 4.93 |  |
|  |  | 60 to 64 | -0.43 | -0.27 | -0.12 | 12.47 | 7.83 | 3.48 |  |
|  |  | 65 to69 | -0.43 | -0.34 | -0.29 | 12.47 | 9.86 | 8.41 |  |
|  |  | ≥70 | -0.52 | -0.40 | -0.23 | 15.08 | 11.6 | 6.67 |  |
| DBP | Medication duration | |  |  |  |  |  |  |  |
|  |  | 29th vs.59th week | -0.27 | - | - | 7.83 | - | - | 0.0003 |
|  |  | 84th vs.112th week | - | -0.22 | - | - | 6.38 | - | 0.0002 |
|  |  | 133rd vs.163th week | - | - | -0.15 | - | - | 4.35 |  |
|  | Gender | |  |  |  |  |  |  | 0.0264 |
|  |  | Male | -0.29 | -0.24 | -0.15 | 8.41 | 6.96 | 4.35 |  |
|  |  | Female | -0.25 | -0.19 | -0.15 | 7.25 | 5.51 | 4.35 |  |
|  | BMI | |  |  |  |  |  |  | 0.8052 |
|  |  | <18.5 | -0.22 | -0.22 | -0.15 | 6.38 | 6.38 | 4.35 |  |
|  |  | 18.5 to 23.9 | -0.27 | -0.22 | -0.15 | 7.83 | 6.38 | 4.35 |  |
|  |  | 24.0 to 27.9 | -0.27 | -0.22 | -0.15 | 7.83 | 6.38 | 4.35 |  |
|  |  | ≥28.0 | -0.31 | -0.22 | -0.14 | 8.99 | 6.38 | 4.06 |  |
|  | Drinking behavior | |  |  |  |  |  |  | 0.0008 |
|  |  | Not drinking | -0.27 | -0.21 | -0.15 | 7.83 | 6.09 | 4.35 |  |
|  |  | Drinking | -0.29 | -0.33 | -0.18 | 8.41 | 9.57 | 5.22 |  |

Sensitivity was defined as the ratio of the BP difference to the temperature difference between the two time points, when the population BP reached its peak (29th, 84th, 133rd week) and bottom (62nd, 112th, 163rd week) during each year of the follow up. This index was compared among different stratifications to examine the modification of the temperature-BP association by other factors. BP indicates blood pressure; SBP, systolic blood pressure; DBP, diastolic blood pressure; BMI, body mass index.

^a^ Estimated by multiplying the absolute value of the “sensitivity” by 29, which was the yearly average temperature change.

^b^ For medication duration, paired t-tests were done between each two neighboring two groups; as to the other factors, repeated measure analyses of variance were used.
